# Relationship of Body Fat and Cardiorespiratory Fitness with Cardiovascular Risk in Chinese Children

**DOI:** 10.1371/journal.pone.0027896

**Published:** 2011-11-16

**Authors:** Pei-gang Wang, Jie Gong, Su-qing Wang, Evelyn O. Talbott, Bo Zhang, Qi-qiang He

**Affiliations:** 1 School of Public Health, Wuhan University, Wuhan, People's Republic of China; 2 Wuhan Center for Disease Control and Prevention, Wuhan, People's Republic of China; 3 Graduate School of Public Health, University of Pittsburgh, Pittsburgh, Pennsylvania, United States of America; 4 School of Public Health, Sun Yat-sen University, Guangzhou, People's Republic of China; Kenya Medical Research Institute - Wellcome Trust Research Programme, Kenya

## Abstract

**Backgrounds/Objectives:**

Cardiorespiratory fitness (CRF) and body fat play an important role in elevated risk for cardiovascular disease (CVD). However, the combined effects of CRF and obesity on metabolic health in Chinese children are unclear. The purpose of this study was to investigate the independent and combined associations between body fat, CRF, and CVD risk in Chinese schoolchildren.

**Methods:**

The study subjects comprised 676 schoolchildren (392 boys and 284 girls, aged 9.6±0.7 yrs old) in Wuhan, China. Their body mass index (BMI), waist circumference (WC), CRF, blood pressure (BP), lipids, glucose, and pubertal status were assessed. Children were categorized into different groups based on their BMI, WC, and CRF using Chinese obesity cut-off points and CRF sex-specific median points. Metabolic Risk Score (MRS) was computed based on the standardized scores of BP, lipids, and glucose.

**Results:**

Multiple linear regression models showed that, in the separate models, body fat was positively associated with MRS while CRF was inversely associated with MRS (p<0.001). However, when assessed simultaneously, only body fat had a significant association with MRS (p<0.001). In general, low-fit children had a lower MRS compared to their counterparts, and a significant difference between the two extreme groups was observed (low CRF and high fat vs. high CRF and low fat, p<0.001).

**Conclusions:**

These findings suggest that both body fat and CRF should be considered when interpreting CVD risk in Chinese children, while body fat may be correlated with CVD risk more than CRF.

## Introduction

Although cardiovascular disease (CVD) is mainly prevalent in adults, a previous study suggested that the precursors of CVD originate in children and adolescents [1]. Risk factors for CVD including hypertension, obesity, physical inactivity, and low physical fitness have been found to track from childhood to adulthood [2].

Epidemiological researches have found that not only obesity but also cardiorespiratory fitness (CRF) plays a pivotal role in the prevalence of CVD. Two longitudinal studies have shown that low CRF was a strong and independent predictor of metabolic health in adults [3], [4]. However, findings from studies in children are inconsistent. One study found a significant association between childhood adiposity and unfavorable metabolic profiles [5], whereas the other suggested that the influence of body fat might be attenuated by high CRF [6].

Over the past 3 decades, China has undergone enormous socioeconomic change. As a consequence, the prevalence of childhood obesity is approaching that of the developed countries [7]. CVD has become the leading cause of death in China, and a national study has revealed that the prevalence of diabetes in Chinese adults aged 20 or older has risen from 2.5% in 1994 to an alarming 9.7% in 2007 [8]. However, the combined effects of CRF and obesity on metabolic health in Chinese children are unclear. The aim of this study was to investigate the independent and combined associations between fitness, body fat, and CVD risk in Chinese schoolchildren.

## Methods

### Study subjects

This cross-sectional study was conducted in Wuhan, China from May to June 2010. Study subjects were recruited into the study in two ways. First, four districts were randomly selected according to location (2 urban and 2 suburban), and second, one primary school was randomly selected in each district. All students in grade 3 and 4 in the schools were invited to participate in the study. Written informed consent was obtained from parents of the children. The study protocol was approved by the Medical Research Ethics Committee of Wuhan University.

### Anthropometric measures and blood pressure

The children's height (standing erect without shoes), weight (in light clothes) and waist circumference (WC) were measured using standard methods. Body mass index (BMI) was calculated by dividing weight (kg) by height squared (m^2^). Pubertal development was assessed by direct observation according to the Tanner stages [9]. Breast development in girls and genital development in boys were used for pubertal classification.

Blood pressure (BP) was measured by a trained technician with all children sitting in an upright position for at least 5 min. Two measurements were taken in the morning and the mean of the two measurements was used for data analysis. The mean arterial pressure (MAP) was calculated as diastolic blood pressure (DBP) + [(systolic blood pressure (SBP) – DBP)/3].

### Cardiorespiratory fitness

CRF was assessed by the 20-meter multistage fitness test (MFT) [10]. This test is a useful measure of cardiorespiratory capacity and has been found to be a validated and reliable field test in children and adolescents [11]. Subjects were asked to run back and forth on a 20-meter course at a pre-determined speed guided by audio signals from a CD player. The running speed was set to increase at 0.5 km/h each minute, from a start speed of 8.5 km/h. Groups of six children were instructed to run at speeds following the audio signal and to complete as many as laps as possible, until they could not cope. The children were stopped when they could not follow the signal any more. Predicted maximum oxygen uptake (VO_2_max) derived from the level (maximal speed) and number of laps in the test was used as a measure of CRF [12].

### Blood samples

Blood samples were taken from the antecubital vein after an overnight fast. Glucose, high-density lipoprotein (HDL), and triglyceride (TG) were analyzed enzymatically at the Wuhan Center for Disease Control and Prevention with a Mairui BS-300 Automatic Analyzer (Mairui High Technologies Corp. Shenzhen, China).

### Metabolic Risk Score

CVD risk factors (MAP, HDL, TG, and glucose) were used to compute the Metabolic Risk Score (MRS). First, each risk factor was standardized as follows: standardized value  =  (value - mean)/standardized deviation. The HDL scores were multiplied by -1 because it is inversely related to metabolic risk. Next, the MRS was calculated as the sum of the four scores. The scores were continuous measures of metabolic risks with higher scores showing a poorer profile.

### Data analysis

Children were categorized as low BMI (normal weight) or high BMI (overweight or obese), and low WC (normal WC) or high WC (high or normal high WC) using Chinese standard age- and gender-specific BMI [13]/WC [14] values. They were also classified into high or low fitness groups based on the gender-specific median values. Cut-off points for boys and girls were 46.85 ml/kg/min and 45.40 ml/kg/min, respectively. Study children were grouped into the following groups based on their BMI, WC and fitness: low BMI (WC) and low fitness, low BMI (WC) and high fitness, high BMI (WC) and low fitness, and high BMI (WC) and high fitness.

The differences in the characteristics between boys and girls were determined using t-test and Chi-square test, where appropriate. As there were no significant interactions between body fat or fitness and sex, all subjects were analyzed together. Multiple linear regression was used to determine the independent or combined effects of BMI, WC, and fitness on MRS. The MRSs in different fitness/fatness groups were compared by analysis of covariance (ANCOVA), controlling for district, age, sex, and pubertal stage. Statistical analyses were performed using the SPSS statistical package (version 13.0; SPSS Inc, Chicago, Ill. USA).

## Results

Of a total of 765 children, 676 (88.4%) aged 9.6 (0.7) years with complete data were included in the final analysis. The children's characteristics stratified by gender are shown in Table 1. Compared to boys, girls show significantly lower height, weight, BMI, WC, DBP, WAP, and fitness.

**Table 1:** Characteristics of the study subjects according to gender.

	Boys(n = 392)	Girls(n = 284)
Age, yr	9.6(0.6)	9.5(0.7)***
Height, cm	137.0(6.5)	135.8(7.7)*
Weight, kg	32.5(7.1)	30.5(7.2)***
BMI, kg/m^2^	17.2(2.8)	16.4(2.7)***
Overweight, n (%)	43 (11.0)	21 (7.4)
Obese, n (%)	31 (7.9)	13 (4.6)
WC, cm	60.1(8.2)	57.5(6.7)***
Normal high WC, n (%)	48 (12.2)	35 (12.3)
High WC, n (%)	37 (9.4)	33 (11.6)
Pubertal Stage I/II, n	391/2	283/1
SBP, mm Hg	92(10)	90(10)
DBP, mm Hg	59(7)	57(8)***
MAP, mmHg	70(7)	68(8)*
TG, mmol/l	1.0(0.6)	1.0(0.5)
HDL, mmol/l	1.2(0.1)	1.2(0.1)
Glucose, mmol/l	4.4(0.4)	4.4(0.4)
Fitness, ml/kg/min	46.5(3.6)	45.0(3.2)***

BMI: Body Mass Index. WC: Waist circumference. SBP: Systolic blood pressure. DBP: Diastolic blood pressure. MAP: Mean arterial pressure. TG: Triglyceride. HDL: High-density lipoprotein.

Values are number (percentage) or mean (SD).

*: p<0.05,

***: p<0.001 for boys vs. girls.

In the separate multiple linear regression models, body fat (BMI/WC) was significantly positively while fitness was inversely associated with MRS. However, when assessed simultaneously, only fatness had a significant association with MRS (p<0.001) (Table 2).

**Table 2:** Multivariate linear regression examining the associations between BMI, WC, fitness and metabolic risk score.

Models		β	95%CI	*p*
1.	BMIscore	0.60	0.45,0.75	<0.001
2.	WCscore	0.66	0.52,0.81	<0.001
3.	CRFscore	-0.53	-0.68,-0.38	<0.001
4.	BMIscore	0.56	0.27,0.85	<0.001
	CRFscore	-0.05	-0.34,0.24	0.736
5.	WCscore	0.63	0.40,0.86	<0.001
	CRFscore	-0.04	-0.27,0.19	0.738

BMI: Body Mass Index. WC: Waist circumference. CRF: Cardiorespiratory fitness.

Adjusted for district, sex, age, and tanner stage.

**Figure 1:**
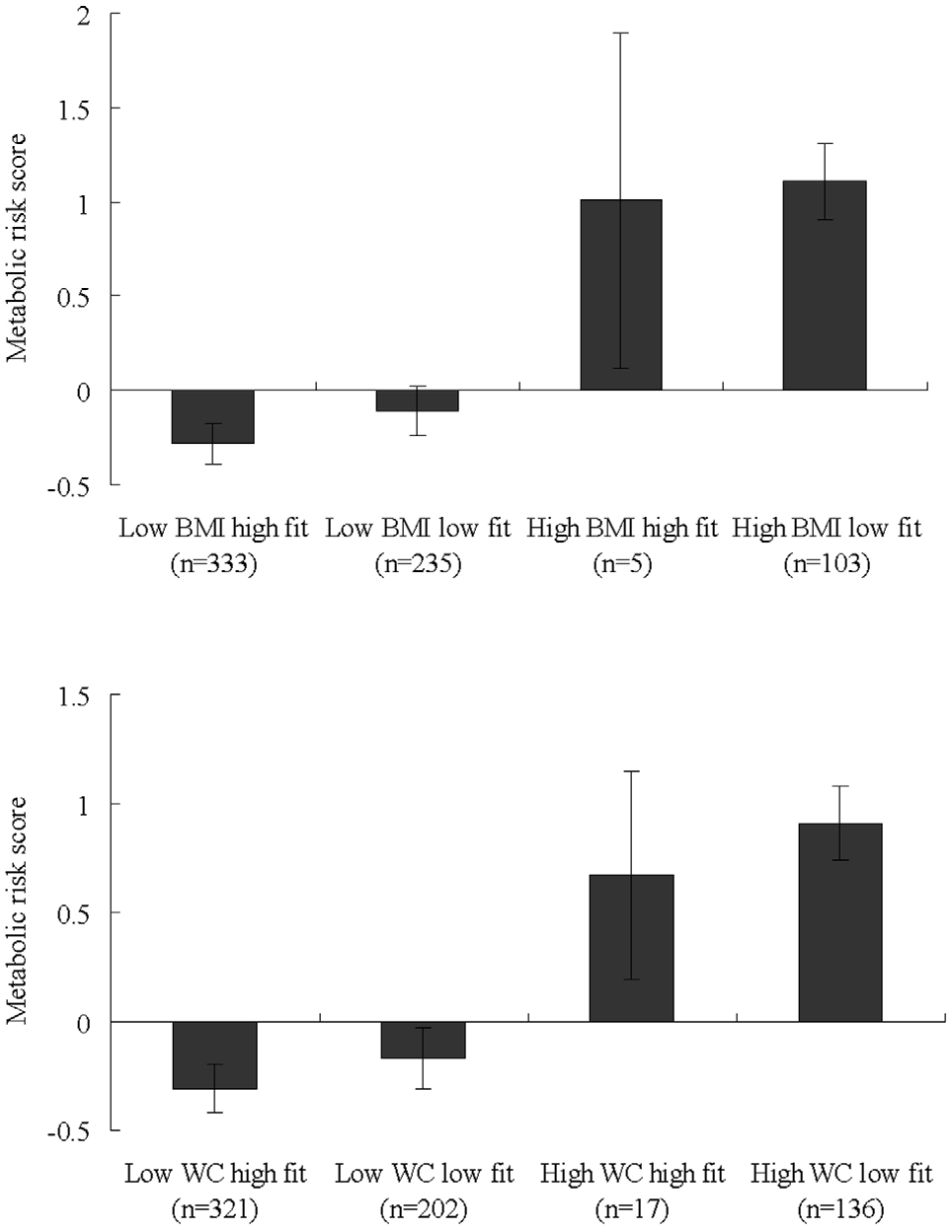
The relationship between fitness, body fat, and MRS in boys and girls.

In general, low-fat children had a lower MRS compared to their counterparts, and a significant difference between the two extreme groups was observed (low CRF and high fatness vs. high CRF and low fatness, p<0.001) (Fig 1).

## Discussion

This study demonstrated that both body fat and CRF should be considered when interpreting CVD risk in schoolchildren. Moreover, fatness correlated with the clustering of CVD risk more than CRF. The present study is the first study that explores the combined effects of body fat and fitness on the metabolic health of Chinese children.

CRF is defined as the overall capacity of the cardiovascular and respiratory systems to provide oxygen during a continuous physical activity to carry out prolonged exercise [15]. There is an important genetic component of CRF [16]. Nevertheless, although it has been suggested that fatness plays an important role in the metabolic profile in adolescents, there is little evidence regarding the influence of fitness on CVD risk in Chinese children. In an earlier prospective study in Chinese schoolchildren, we showed a strong negative association between CRF and BMI and weight gain [17]. Results from the present study provide further evidence of a beneficial effect of fitness for reducing metabolic health risks. Taken together, these findings highlight the significance of improving CRF for preventing obesity and lowering the risk for CVD in Chinese children.

BMI has been widely adopted in epidemiological studies as a measure of adiposity; however, it has been criticized as being an inaccurate measurement [18]. Moreover, one previous study has used the BMI median split instead of health-related cut-off points to classify study subjects as high or low fatness [19]. The paper overcomes this limitation by using Chinese overweight/obesity definitions. In addition, two different indicators of adiposity, BMI (total adiposity) and WC (abdominal adiposity), were used in the present study. WC is a strong predictor of visceral fat, and evidence has shown significant associations between WC and CVD risk in children in their first decade of life [20]. Results from current study regarding the relationships of BMI and WC with CVD risk are in accordance with previous studies demonstrating a great influence of body fat on metabolic health [5], [6].

Several studies have suggested that high levels of CRF attenuate the deleterious consequences ascribed to high fatness [6], [21]. In the present study, although high fit children had lower MRSs than the low fit children within the same BMI group, the differences were statistically insignificant. As no standard definition for low CRF exists in Chinese children, the classification of high and low fitness was based on median cut-points. Healthy children might have been categorized into the low fitness group; thus, bias towards the null due to misclassification might explain the results. Therefore, future studies using evidence-based cut-off points for fitness are required to draw more conclusions on the interactive effects of fitness and fatness on metabolic health in Chinese schoolchildren.

Boys had significantly higher fitness levels than girls in this study, which are in line with our recent finding [17] and most studies of Caucasian children [22], [23]. Nevertheless, the results show that the influence of body fat and fitness on children's CVD risk was independent of gender. Our findings lend support to several previous investigations [21], [24]. In contrast, Elsenmann et al. reported that high fit girls had significantly lower blood pressure than their counterparts in the same fatness group, while the differences in boys were insignificant [25], whereas Cummings et al. found a significant relationship between fitness and insulin resistance in boys but not girls [26]. The reasons for the conflicting findings remain unclear. Genetic predisposition and several other environmental and biological factors might all be involved in the interplay among fitness, body fat, and CVD risk.

In the present study, CRF was not significantly associated with MRS when body fat and CRF were simultaneously assessed. The possible reason may be that we have expressed CRF (peak VO_2max_) in relation to body weight because oxygen and energy needs differ relative to size. Therefore, there is a risk of creating statistical artifacts when adiposity was adjusted for because it is a part of the component. On the other hand, some researchers suggested that fitness and fatness are associated with MRS through different pathways [27] or fatness only partially mediates the effect of fitness on MRS [28].

Limitations of this study should be recognized when interpreting the results. First, the cross-sectional design limits our ability to establish a cause-effect relationship of fitness and fatness on metabolic health. Data obtained from a prospective study of these children may help to verify the findings. Second, fitness was assessed indirectly. Although performance on the test may be affected by motivation, previous studies have shown that this test is reliable, and the predicted CRF is highly correlated with the measured values in the laboratory [11]. Third, generalization of data collected from this study to the provincial or the national level must be made with caution.

In summary, we have provided evidence of significant effects of body fat and fitness on metabolic health in Chinese schoolchildren. Given the rapidly increasing prevalence of obesity and concerns for adverse CVD risk factors in Chinese children, therapeutic and health promotion interventions aimed at improving fitness and preventing childhood obesity could result in substantial public health benefit.
